# Circulating osteocalcin as a bone-derived hormone is inversely correlated with body fat in patients with type 1 diabetes

**DOI:** 10.1371/journal.pone.0216416

**Published:** 2019-05-03

**Authors:** Yuichi Takashi, Masashi Ishizu, Hiroyasu Mori, Kazuyuki Miyashita, Fumie Sakamoto, Naoto Katakami, Taka-aki Matsuoka, Tetsuyuki Yasuda, Seiichi Hashida, Munehide Matsuhisa, Akio Kuroda

**Affiliations:** 1 Diabetes Therapeutics and Research Center, Institute of Advanced Medical Sciences, Tokushima University, Tokushima, Japan; 2 Department of Hematology, Endocrinology and Metabolism, Tokushima University Graduate School of Biomedical Sciences, Tokushima, Japan; 3 Department of Metabolic Medicine, Osaka University Graduate School of Medicine, Osaka, Japan; 4 Department of Diabetes and Endocrinology, Osaka Police Hospital, Osaka, Japan; 5 Institute for Health Sciences, Tokushima Bunri University, Tokushima, Japan; Ehime University Graduate School of Medicine, JAPAN

## Abstract

The objective of the present study was to investigate the correlations between serum undercarboxylated osteocalcin (ucOC) or osteocalcin (OC) concentrations and %body fat, serum adiponectin and free-testosterone concentration, muscle strength and dose of exogenous insulin in patients with type 1 diabetes. We recruited 73 Japanese young adult patients with childhood-onset type 1 diabetes. All participants were receiving insulin replacement therapy. The correlations between logarithmic serum ucOC or OC concentrations and each parameter were examined. Serum ucOC and OC concentrations were inversely correlated with %body fat (r = -0.319, P = 0.007; r = -0.321, P = 0.006, respectively). Furthermore, multiple linear regression analyses were performed to determine whether or not serum ucOC or OC concentrations were factors associated with %body fat. Serum ucOC and OC concentrations remained significant factors even after adjusting for gender, HbA1c, body weight-adjusted total daily dose of insulin and duration of diabetes (β = -0.260, P = 0.027; β = -0.254, P = 0.031, respectively). However, serum ucOC and OC concentrations were not correlated with serum adiponectin or free-testosterone concentrations, muscle strength or dose of exogenous insulin. In conclusion, our study demonstrates the inverse correlation between serum ucOC or OC concentrations and body fat in patients with type 1 diabetes.

## Introduction

Bones perform several functions, such as supporting the body, protecting the internal organs and central nervous system and contributing to hematopoiesis. Recently, it has been reported that the skeleton also functions as an endocrine organ and systemically regulates the functions of other organs [1].

Several bone-derived hormones have been defined, including fibroblast growth factor 23 and lipocalin 2, among others [2–5]. Osteocalcin (OC), another such hormone, is reported to affect glucose and energy metabolism [6]. It is produced by osteoblasts and acts as a bone matrix protein. The serum level of OC was reported to be sustained after adulthood, and the reference value of serum OC concentrations is 8.4–33.1 ng/ml in males, 7.8–30.8 ng/ml in premenopausal females and 14.2–54.8 ng/ml in postmenopausal females [7]. Undercarboxylated osteocalcin (ucOC) is considered to be the active form of circulating OC, exerting endocrine functions according to experimental studies [8, 9]. However, whether or not ucOC is the active form in humans as well is unclear. The reference value of the serum ucOC concentrations is <4.5 ng/ml in general, although Shiraki et al. reported that the mean serum ucOC concentrations was 3.0 ng/ml in their Japanese cohort study [10].

Circulating ucOC binds to the G protein-coupled receptor GPRC6A on pancreatic β-cells and stimulates insulin secretion in animal models [11, 12]. In addition, the serum ucOC concentrations were shown to correlate with the insulin sensitivity in patients with type 2 diabetes [13, 14]. It was also indicated that higher serum ucOC concentrations were correlated with a reduction in the diabetes risk in community-dwelling populations [15, 16], and ucOC enhanced the β-cell function in human islets from cadaveric donors [17]. Furthermore, the production of ucOC is regulated by insulin signaling via insulin receptor on the osteoblasts of a mouse model [18, 19]. Therefore, a positive feedback loop exists between ucOC and insulin.

We previously investigated the correlation between the serum ucOC concentrations and secretory ability of insulin in patients with type 2 diabetes [20]. ucOC was shown to correlate positively with the change in the C-peptide response in the glucagon loading test and the meal tolerance test [20]. It has also been reported that OC was correlated with a reduced fat mass and increased serum adiponectin levels, serum testosterone levels and muscle strength in OC knockout mice [6, 21–23]. In addition, serum ucOC concentrations were shown to be correlated inversely with fat mass and positively correlated with serum adiponectin and serum free-testosterone concentrations in patients with type 2 diabetes [24–26].

As mentioned above, the effects of ucOC on glucose and energy metabolism may promote the pathophysiology and complications of diabetes. Evidence supporting the clinical significance of ucOC concerning type 2 diabetes is gradually accumulating, and many clinical studies have provided supportive experimental results. However, there are few equivalent data in patients with type 1 diabetes [27–29]. Experimental results suggest that the production of ucOC is regulated by exogenous insulin injection in patients with type 1 diabetes whose endogenous insulin secretion has been depleted. Furthermore, the metabolically beneficial effects of ucOC, such as its effects of reducing fat mass, increasing adiponectin, increasing testosterone levels and increasing exercise capacity, are also found in patients with type 1 diabetes.

Since type 2 diabetes is a heterogenous disease, we focused on young adult patients with type 1 diabetes to assess the influence of the exogenous insulin dose on the ucOC and OC concentrations and to determine its metabolic effects clearly. We therefore investigated the correlations between serum ucOC or OC concentrations and clinical characteristics in young adult patients with type 1 diabetes in this study.

## Subjects and methods

### Study design

The study protocol was approved by the research ethics committee of Tokushima University, Tokushima, Japan (#2281–7), and was registered as a clinical trial (UMIN000020901).

We recruited 77 young patients with type 1 diabetes who had undergone medical checkups for diabetic complications at Osaka University Hospital and Osaka Police Hospital in July and August 2017. These medical checkups were annually conducted by Advisory Doctors of Osaka Association for Diabetes Education and Care. Each patient provided their written informed consent.

We excluded 4 patients who were <20 years old, as the serum OC concentrations is known to be higher in adolescents than in adults [7]. We therefore analyzed the data from 73 subjects. The median age was 35 years old, and 21 males (30.1%) and 52 females (69.9%) were included. All participants were treated by diabetologists and were receiving insulin injections at least four times daily or with continuous subcutaneous insulin infusion. Exogenous injected doses of insulin were investigated for more than three days and defined as an average amount of insulin. We enrolled only typical childhood-onset type 1 diabetes patients in this study, excluding those with slowly progressive type 1 diabetes. As vitamin K insufficiency is known to increase the serum ucOC concentration, participants with eating disorders, anemia, hemorrhagic diathesis and hepatic disorders were not included in this study.

### Measurement procedures

Blood samples were obtained during the medical checkups. Glycated hemoglobin (HbA1c) was measured using high-performance liquid chromatography. Based on the serum creatinine concentrations (Cr), the estimated glomerular filtration rate (eGFR) was calculated according to the equation of the Japanese Society of Nephrology; eGFR (mL/min/1.73 m^2^) = 194 × Cr^-1.094^ × age^-0.287^ (× 0.739 if female). Serum ucOC concentrations (electro-chemiluminescence immunoassay; ECLIA), serum OC concentrations (ECLIA), serum adiponectin concentrations (latex agglutination turbidimetric immunoassay; LA) and serum free-testosterone concentrations (in males) (radio immunoassay; RIA) were examined (SRL, Tokyo, Japan).

Our measurement of OC evaluated the total OC levels, including the ucOC. The coefficient of variation (CV) of osteocalcin measurement was <15%. The measurement of ucOC evaluated OC molecules with two uncarboxylated glutamic acid residues at positions 21 and 24, but not the carboxylated one. The CV of ucOC measurement was <10%. OC and ucOC were measured by direct immunoassays with commonly used commercially available antibodies against OC and ucOC, respectively [30, 31].

The protocol for evaluating the body composition and muscle strength was described previously [32]. In brief, the body composition—including the body fat and skeletal muscle—were analyzed with the In Body bioelectrical impedance analyzer (Bio Space, Seoul, Korea). The total fat mass and lean body mass evaluated by this device were validated by comparing the findings with those of dual-energy X-ray absorptiometry [33, 34]. However, the bioelectrical impedance analyzer cannot distinguish visceral and subcutaneous fat mass. The skeletal muscle mass index (SMI) was calculated by dividing the extremity skeletal muscle by the height-squared. The grip strength and knee extension strength were measured using handheld dynamometers (T.K.K.5401, Takei Scientific Instruments, Tokyo, Japan; μ-tus F-100, ANIMA, Tokyo, Japan). Gait speed was assessed by recording the total time it took to walk five meters.

### Statistical analyses

Continuous variables with normal or non-normal distributions were described as the mean ± standard deviation or median (Q1, Q3), respectively. Categorical variables were reported as percentages (%). Statistical analyses were performed using the SPSS 24 software program (IBM Japan, Tokyo, Japan). Because the serum ucOC and OC concentrations did not show normal distributions, the data were also analyzed using log-transformed ucOC and OC values. The Pearson’s correlation coefficient, two-way analysis of variance and multiple linear regression analysis were used. A P value of < 0.05 was considered to be statistically significant.

## Results

The characteristics of the participants are shown in Table 1. The mean duration of diabetes was 25 ± 7 years, and the median body mass index (BMI) was 22.9 kg/m^2^. Concerning their insulin regimen, the rates of participants receiving multiple daily injections of insulin (MDI) and continuous subcutaneous insulin injection (CSII) were 46.6% and 53.4%, respectively. The median total daily dose of insulin (TDD) was 40.0 units, and the body weight-adjusted TDD (TDD/kg) was 0.65 units/kg. The mean basal and bolus dose of insulin were 14.8 units (Basal) and 22.9 units (Bolus), respectively, and the mean body weight-adjusted basal and bolus dose of insulin were 0.25 units/kg (Basal/kg) and 0.39 units/kg (Bolus/kg), respectively. The mean HbA1c was 7.4% ± 1.0%, and the eGFR was 92.9 ± 14.1 ml/min/1.73 m^2^. The median albuminuria was 3.9 mg/gCr, and no one met the diagnostic criteria of chronic kidney disease.

**Table 1:** Clinical characteristics of the participants.

		Reference range
Gender (male/female) (%)	30.1/69.9	
Age (years)	35 (31, 39)	
Duration of diabetes (years)	25.7 ± 7.3	
BMI (kg/m^2^)	22.9 (21.5, 25.1)	18.5–25.0
Insulin regimen (MDI/CSII) (%)	46.6/53.4	
TDD (units)	40.0 (31.3, 50.5)	
TDD/kg (units/kg)	0.65 (0.52, 0.77)	
Basal (units)	14.8 (11.4, 18.7)	
Basal/kg (units/kg)	0.25 (0.19, 0.29)	
Bolus (units)	22.9 (18.0, 34.5)	
Bolus/kg (units/kg)	0.39 (0.32, 0.53)	
HbA1c (%)	7.4 ± 1.0	4.6–6.2
eGFR (ml/min/1.73 m^2^)	92.9 ± 14.1	> 60
Albuminuria (mg/gCr)	3.9 (2.9, 6.0)	< 30
Retinopathy (NDR/NPDR/PDR/NA) (%)	42.5/50.7/5.5/1.4	
%Body fat (%)	26.8 ± 7.2	Male: 15–20 Female: 20–25
Adiponectin (μg/ml)	12.9 (9.9, 17.4)	> 4.0
Free-testosterone (pg/ml) (in males)	10.9 (8.6, 13.2)	20–29 years old: 7.6–23.8 30–39 years old: 6.5–17.7 40–49 years old: 4.7–21.6
SMI (kg/m^2^)	6.7 (6.3, 7.5)	Male: > 7.0 Female: > 5.7
Grip strength (kg)	30.2 (23.0, 38.4)	Male: > 26.0 Female: > 18.0
Knee extension strength (kg)	20.6 ± 5.9	> 0.3
Gait speed (m/s)	1.34 ± 0.22	> 0.8
ucOC (ng/ml)	3.3 (2.4, 4.7)	< 4.5
OC (ng/ml)	14.8 (11.7, 18.5)	Male: 8.4–33.1 Female: 7.8–30.8 (premenopausal)
Use of drugs		
ACE inhibitors/ARBs (%)	16.4	
CCBs (%)	4.1	
Statins (%)	8.2	
EPAs (%)	5.5	

BMI, body mass index; MDI, multiple daily injections of insulin; CSII, continuous subcutaneous insulin infusion; TDD, total daily dose of insulin; TDD/kg, body weight-adjusted total daily dose of insulin; Basal, basal dose of insulin; Basal/kg, body weight-adjusted basal dose of insulin; Bolus, bolus dose of insulin; Bolus/kg, body weight-adjusted bolus dose of insulin; HbA1c, glycated hemoglobin; eGFR, estimated glomerular filtration rate; NDR, no diabetic retinopathy; NPDR, non-proliferative diabetic retinopathy; PDR, proliferative diabetic retinopathy; SMI, skeletal muscle mass index; ucOC, undercarboxylated osteocalcin; OC, osteocalcin; ACE, angiotensin-converting enzyme; ARB, angiotensin receptor blocker; CCB, calcium channel blocker; EPA, eicosapentaenoic acid.

Concerning diabetic retinopathy, the rates of participants with no diabetic retinopathy (NDR), non-proliferative diabetic retinopathy (NPDR) and proliferative diabetic retinopathy (PDR) were 42.5%, 50.7% and 5.5%, respectively. In addition, no one met the Asian diagnostic criteria of sarcopenia [35]. The median serum ucOC and OC concentrations were 3.3 ng/ml and 14.8 ng/ml, respectively. No participants used anti-diabetic drugs besides insulin, and only a small number used anti-hypertensive or anti-hyperlipidemic drugs.

**Fig 1:**
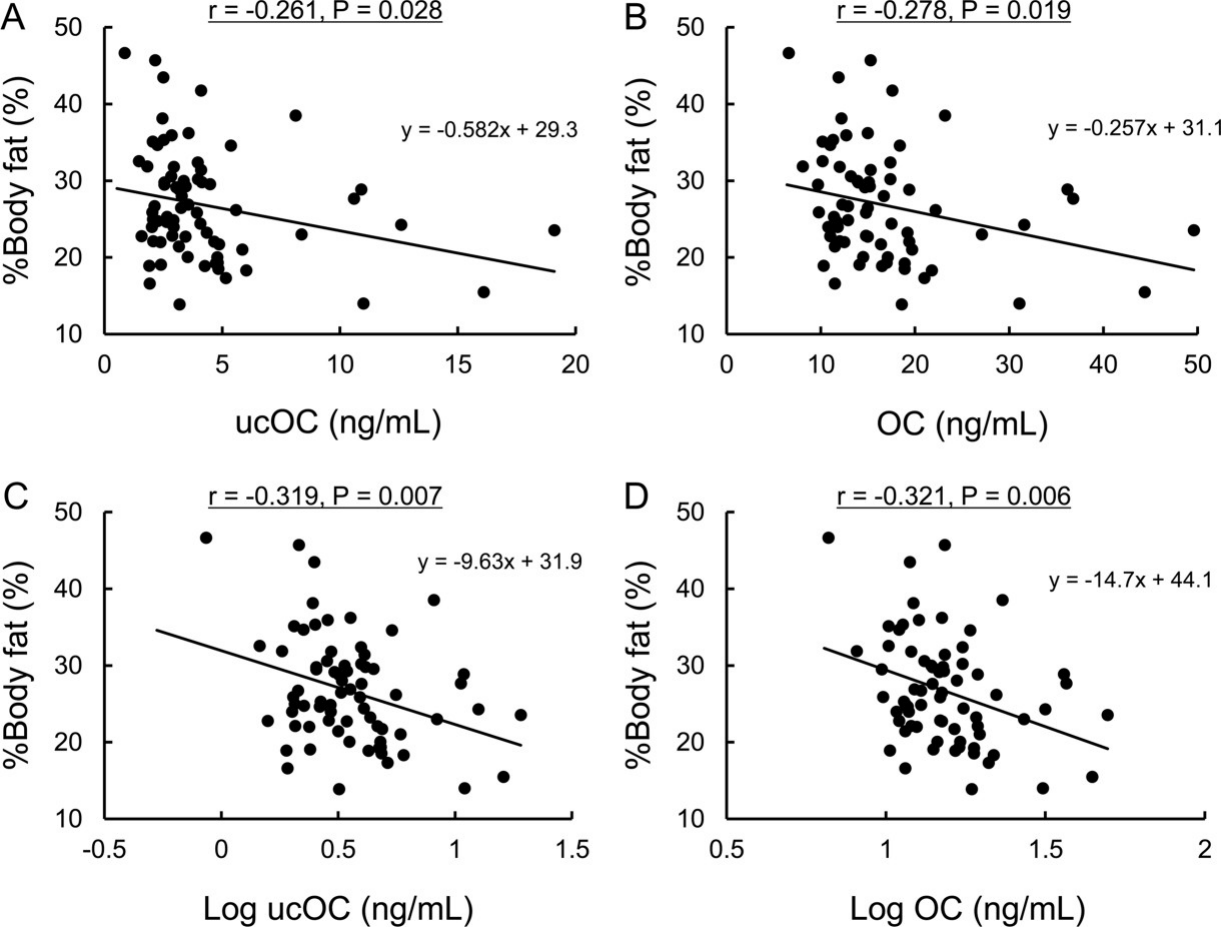
UcOC and OC were inversely correlated with %body fat. The correlation between %body fat and ucOC (A), OC (B), log ucOC (C) and log OC (D). Pearson’s correlation coefficients (r), P values and regression equations are shown.

UcOC and OC were not correlated with the gender, age, duration of diabetes, BMI, HbA1c or eGFR and were only significantly inversely correlated with %body fat (ucOC: r = -0.261, P = 0.028; OC: r = -0.278, P = 0.019; log ucOC: r = -0.319, P = 0.007; log OC: r = -0.321, P = 0.006) (Table 2 and Fig 1). UcOC and OC were not correlated with the insulin regimen or TDD/kg, including Basal/kg and Bolus/kg (Table 2). We reanalyzed the correlation between the log ucOC or OC and dose of exogenous insulin separately for MDI and CSII (S1A Table). However, we noted no correlations between log ucOC or OC and the dose of exogenous insulin in the unadjusted model or after adjusting for age and gender (S1B Table). In addition, there were no correlations between ucOC or OC and the serum adiponectin or serum free-testosterone concentrations (in males), SMI, grip strength, knee extension strength or gait speed (Table 2). The log ucOC and log OC were correlated each other (r = 0.946, P < 0.001).

**Table 2:** Correlations between logarithmic serum ucOC and OC concentrations and each parameter in univariate models.

	ucOC (ng/ml)		OC (ng/ml)		Log ucOC (ng/ml)		Log OC (ng/ml)	
	r	P	r	P	r	P	r	P
Age (years)	-0.157	0.184	-0.178	0.132	-0.186	0.114	-0.193	0.102
Duration of diabetes (years)	-0.174	0.140	-0.176	0.137	-0.138	0.243	-0.143	0.229
BMI (kg/m^2^)	-0.184	0.120	-0.176	0.137	-0.204	0.084	-0.185	0.117
TDD (units)	-0.032	0.788	-0.030	0.798	0.012	0.918	-0.009	0.942
TDD/kg (units/kg)	0.061	0.607	0.057	0.635	0.089	0.456	0.061	0.611
Basal (units)	-0.040	0.738	-0.030	0.803	0.011	0.923	-0.002	0.984
Basal/kg (units/kg)	0.040	0.737	0.053	0.658	0.065	0.584	0.061	0.608
Bolus (units)	-0.021	0.860	-0.024	0.838	0.010	0.933	-0.010	0.931
Bolus/kg (units/kg)	0.049	0.680	0.036	0.762	0.067	0.575	0.036	0.764
HbA1c (%)	-0.086	0.473	-0.116	0.334	-0.057	0.635	-0.127	0.288
eGFR (ml/min/1.73 m^2^)	-0.203	0.086	-0.231	0.050	-0.103	0.387	-0.168	0.155
Albuminuria (mg/gCr)	0.074	0.535	0.102	0.389	0.028	0.816	0.085	0.475
%Body fat (%)	-0.261	0.028*	-0.278	0.019*	-0.319	0.007*	-0.321	0.006*
Adiponectin (μg/ml)	-0.078	0.511	-0.060	0.617	-0.140	0.237	-0.070	0.554
Free-testosterone (pg/ml) (in males)	0.081	0.719	0.104	0.644	0.074	0.743	0.079	0.726
SMI (kg/m^2^)	-0.060	0.622	-0.043	0.719	-0.061	0.613	-0.017	0.885
Grip strength (kg)	0.017	0.887	0.030	0.800	0.146	0.222	0.124	0.298
Knee extension strength (kg)	0.014	0.908	0.055	0.642	0.103	0.386	0.116	0.330
Gait speed (m/s)	-0.055	0.649	0.005	0.969	0.009	0.943	0.084	0.483

* Statistically significant (P < 0.05).

ucOC, undercarboxylated osteocalcin; OC, osteocalcin; BMI, body mass index; MDI, multiple daily injection of insulin; CSII, continuous subcutaneous insulin infusion; TDD, total daily dose of insulin; TDD/kg, body weight-adjusted total daily dose of insulin; Basal, basal dose of insulin; Basal/kg, body weight-adjusted basal dose of insulin; Bolus, bolus dose of insulin; Bolus/kg, body weight-adjusted bolus dose of insulin; HbA1c, glycated hemoglobin; eGFR, estimated glomerular filtration rate; SMI, skeletal muscle mass index.

**Fig 2:**
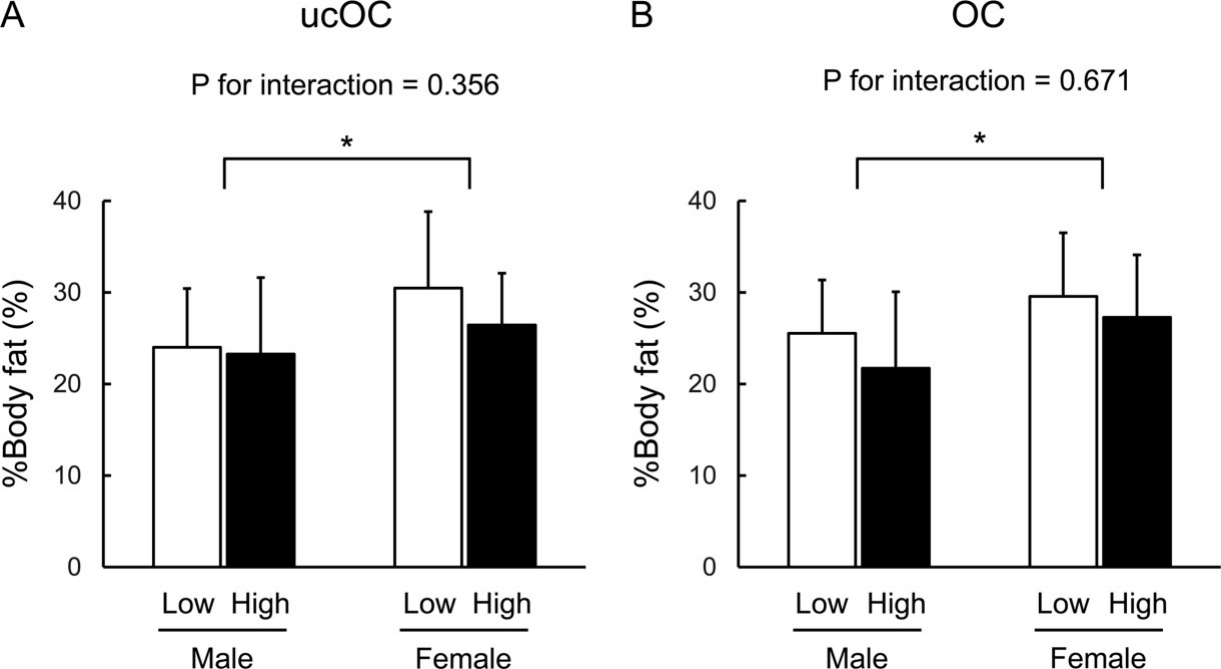
Two-way ANOVA for %body fat concerning factors of gender and categorical variables of ucOC or OC. There were significant differences in %body fat by gender (P = 0.0086, in the ucOC model; P = 0.0097, in the OC model). In contrast, there were no significant differences in %body fat by categorical variables of ucOC or OC (P = 0.185; P = 0.094, respectively). In addition, there were no significant interactions between gender and ucOC or OC (P = 0.356; P = 0.671, respectively). * Statistically significant (P < 0.05).

We further performed a two-way analysis of variance for %body fat concerning the factors of gender and categorical variables of ucOC or OC divided at the median serum ucOC or OC concentration. There were significant differences in %body fat by gender (P = 0.0086, in the ucOC model; P = 0.0097, in the OC model) (Fig 2). In contrast, there were no significant differences in %body fat by the categorical variable of ucOC and OC (P = 0.185; P = 0.094, respectively). In addition, there were no significant interactions between gender and ucOC or OC (P = 0.356; P = 0.671, respectively) (Fig 2).

Next, multiple linear regression analyses were performed to identify the serum ucOC or OC concentration as a factor associated with %body fat. Before the analysis, we confirmed that the values of %body fat showed a normal distribution curve (P > 0.200, Kolmogorov-Smilnov test). In model 1 adjusted for gender, ucOC and OC remained significant (ucOC: β = -0.270, P = 0.019; OC: β = -0.279, P = 0.016; log ucOC: β = -0.303, P = 0.008; log OC: β = -0.300, P = 0.009) (Table 3). In model 2, which was further adjusted for HbA1c, ucOC and OC also remained significant (ucOC: β = -0.233, P = 0.039; OC: β = -0.234, P = 0.039; log ucOC: β = -0.252, P = 0.027; log OC: β = -0.246, P = 0.032) (Table 3). Finally, in model 3, which was further adjusted for TDD/kg and duration of diabetes, ucOC and OC still remained significant (ucOC: β = -0.240, P = 0.038; OC: β = -0.243, P = 0.038; log ucOC: β = -0.260, P = 0.027; log OC: β = -0.254, P = 0.031) (Table 3).

**Table 3:** Multiple linear regression analyses between %body fat and logarithmic serum ucOC or OC concentration.

	ucOC (ng/ml)		Log ucOC (ng/ml)	
	β	P	β	P
Model 1	-0.270	0.019*	-0.303	0.008*
Model 2	-0.233	0.039*	-0.252	0.027*
Model 3	-0.240	0.038*	-0.260	0.027*
	OC (ng/ml)		Log OC (ng/ml)	
	β	P	β	P
Model 1	-0.279	0.016*	-0.300	0.009*
Model 2	-0.234	0.039*	-0.246	0.032*
Model 3	-0.243	0.038*	-0.254	0.031*

Model 1: adjusted for gender. Model 2: model 1 + HbA1c. Model 3: mode 2 + TDD/kg and duration of diabetes

* Statistically significant (P < 0.05).

β, standard partial regression coefficient; ucOC, undercarboxylated osteocalcin; OC, osteocalcin; HbA1c, glycated hemoglobin; TDD/kg, body weight-adjusted total daily dose of insulin.

## Discussion

In the present study, we evaluated the serum ucOC or OC concentrations and clinical characteristics in young adult patients with long standing type 1 diabetes. We found an inverse correlation of ucOC and OC with %body fat, in accordance with the results in experimental models [6, 11]. Previous experimental studies have shown that OC knockout mice have an increased fat mass [6], and continuous injection of ucOC was shown to decrease the fat mass [11]. In addition, serum ucOC concentrations were shown to be inversely correlated with %trunk fat in patients with type 2 diabetes [24]. We observed this correlation between ucOC and fat mass in patients with type 1 diabetes as well. Although ucOC was considered to be the active form of OC in experimental findings, serum OC concentrations were also inversely correlated with %body fat, similar to ucOC, in this study.

The assay for OC detects both the carboxylated and uncarboxylated forms, reflecting the total OC. We measured the amount of undercarboxylated osteocalcin retaining the carboxylated residue at position 17. Theoretically, the ucOC level should be one-quarter of the total OC amount. Indeed, the mean serum ucOC level (4.2 ng/ml) was roughly one-quarter of the serum level of total OC (16.5 ng/ml) in our cohort. In addition, the serum ucOC and OC levels were highly correlated (r = 0.946, P < 0.001). Previous experimental models have shown that insulin signaling enhances both the production of OC from osteoblasts and the conversion of OC into ucOC by osteoclasts, so ucOC and OC are balanced. Therefore, we were able to detect a significant correlation of %body fat with both ucOC and OC.

In addition, we performed a multiple linear regression analysis involving both ucOC and OC, simultaneously. As a result, both ucOC and OC lost significance. However, they were suggested to have multicollinearity (variance inflation factor [VIF] > 10). As mentioned above, we were unable to distinguish the significance of ucOC and OC in the present study. As such, whether or not ucOC is the active form of OC in humans remains unclear. In addition, the receptor of ucOC on the adipocytes to reduce fat mass has not yet been identified [8], and ucOC may exert no direct effects on adipose tissue. Thus, further studies are necessary in order to clarify the precise effects of ucOC or OC on adipocytes.

The BMI was not found to be correlated with ucOC or OC in the univariate model (Table 2). While the BMI was significantly correlated with logarithmic ucOC (but not OC) after adjusting for each factor (S2 Table), this correlation with BMI was weaker than that with %body fat. Although the BMI was correlated with %body fat (r = 0.701, P < 0.001), we consider this to be because the BMI mainly reflects not only the fat mass but also skeletal muscle. Neither ucOC nor OC correlated with the SMI even after adjusting for each factor. In general, gender, age, blood glucose level and insulin affect fat mass; however, serum ucOC and OC concentrations remained significantly inversely correlated with %body fat even after adjusting for these factors. The bone-derived hormones ucOC and OC may thus prevent weight gain in patients with type 1 diabetes in contrast to the administration of insulin therapy, etc. Taken together, these present and previous findings suggest that ucOC may be a viable target for the development of new drugs for diabetes.

In contrast to previous reports of experimental studies, we failed to observe any correlations between serum ucOC or OC concentrations and the dose of exogenous insulin, serum adiponectin concentrations, serum free-testosterone concentrations in males or muscle strength, such as the SMI, grip strength, knee extension strength or gait speed even after adjusting for each factor. In detail, first, while we did observe a correlation between serum ucOC or OC concentrations and %body fat, we did not observe any correlation between serum ucOC or OC concentrations and serum adiponectin concentration. This may be due to the substantial increase in the serum adiponectin level in patients with type 1 diabetes treated by insulin therapy [36]. Second, we also failed to observe any marked relationship between serum ucOC or OC concentrations and serum free-testosterone concentrations in males, possibly because the participants of our study were relatively young and did not meet the diagnostic criteria of late-onset hypogonadism. Third, we observed no relationships between serum ucOC or OC concentrations and the SMI, grip strength, knee extension strength or gait speed, likely because our participants were relatively young and did not meet the diagnostic criteria of sarcopenia. While a previous report efficiently evaluated the endurance of muscles in a mouse model [23], it is difficult to evaluate the physical endurance of patients. At least ucOC or OC were not correlated with muscular parameters used as a diagnosis of sarcopenia at clinical settings in this study [35]. Fourth, we were unable to confirm a relationship between serum ucOC or OC concentrations and the dose of exogenous insulin. Based on our experimental results, we hypothesized that the serum ucOC concentrations were regulated by exogenous insulin in patients with type 1 diabetes. However, which factors determine the serum concentrations of ucOC in patients with type 1 diabetes remains unclear.

The present study had several limitations. First, this was a cross-sectional study with a small sample size without a control group. We were therefore unable to prove the causality or indicate the direction of the association concerning ucOC or OC. Second, we were unable to consider behavioral or lifestyle factors, including meals or exercise regimens, or the influence of drug usage, such as anti-hypertensive or lipidemic drugs. Third, we were unable to examine the vitamin K intake or plasma vitamin K concentrations, which can affect serum ucOC concentrations, and bone turnover markers, including 25-hydroxyvitamin D and parathyroid hormone, or bone mineral densities. Further longitudinal or clinical manipulation studies will be necessary to determine the significance of ucOC or OC on glucose and energy metabolism in type 1 diabetes.

In conclusion, our study showed the inverse correlation between serum ucOC or OC concentrations and body fat in patients with type 1 diabetes, as previously reported in experimental models and patients with type 2 diabetes. One of the beneficial effects of ucOC or OC was also observed in patients with type 1 diabetes.

## Supporting information

S1 Table Correlations between dose of exogenous insulin and serum ucOC or OC concentration separately for MDI and CSII. (DOCX)

S2 Table Multiple linear regression analysis between BMI and logarithmic serum ucOC or OC concentration. (DOCX)

S1 File Minimal data set of this study. (XLSX)
