# Supplementary material for: Circulating osteocalcin as a bone-derived hormone is inversely correlated with body fat in patients with type 1 diabetes
Source: PLoS One. 2019 May 3;14(5):e0216416. doi: 10.1371/journal.pone.0216416 (PMC6499427; doi:10.1371/journal.pone.0216416)
Supplement: S1 Table — (DOCX) [file pone.0216416.s001.docx]

**S1 Table. Correlations between dose of exogenous insulin and serum ucOC or OC concentration separately for MDI and CSII.**

**A. Characteristics of the participants.**

|  | MDI | CSII |
| --- | --- | --- |
| TDD (units) | 44.0 (35.4, 60.8) | 33.0 (29.9, 43.7) |
| TDD/kg (units/kg) | 0.76 ± 0.23 | 0.61 ± 0.15 |
| Basal (units) | 16.0 (13.0, 25.2) | 14.6 ± 0.24 |
| Basal/kg (units/kg) | 0.26 (0.21, 0.33) | 0.24 ± 0.07 |
| Bolus (units) | 27.0 (21.0, 45.3) | 21.7 (16.9, 38.3) |
| Bolus/kg (units/kg) | 0.49 ± 0.19 | 0.35 (0.31, 0.43) |

**B. Correlations between** **TDD/kg and logarithmic serum ucOC or OC concentration.**

|  | MDI | | | | CSII | | | |
| --- | --- | --- | --- | --- | --- | --- | --- | --- |
|  | Log ucOC | | Log OC | | Log ucOC | | Log OC | |
|  | β | P | β | P | β | P | β | P |
| Model 1 | 0.099 | 0.579 | 0.141 | 0.425 | -0.038 | 0.817 | -0.109 | 0.508 |
| Model 2 | 0.034 | 0.873 | 0.020 | 0.921 | -0.060 | 0.715 | -0.127 | 0.448 |

Model 1: unadjusted

Model 2: adjusted for age and gender

β, standard partial regression coefficient; ucOC, undercarboxylated osteocalcin; OC, osteocalcin; MDI, multiple daily injection of insulin; CSII, continuous subcutaneous insulin infusion; TDD, total daily dose of insulin; TDD/kg, body weight adjusted total daily dose of insulin; Basal, basal dose of insulin; Basal/kg, body weight adjusted basal dose of insulin; Bolus, bolus dose of insulin; Bolus/kg, body weight adjusted bolus dose of insulin.
