# Supplementary material for: Circulating osteocalcin as a bone-derived hormone is inversely correlated with body fat in patients with type 1 diabetes
Source: PLoS One. 2019 May 3;14(5):e0216416. doi: 10.1371/journal.pone.0216416 (PMC6499427; doi:10.1371/journal.pone.0216416)
Supplement: S2 Table — (DOCX) [file pone.0216416.s002.docx]

**S2 Table. Multiple linear regression analysis between BMI and logarithmic serum ucOC or OC concentration.**

|  | Log ucOC (ng/ml) | |
| --- | --- | --- |
|  | β | P |
| Model 1 | -0.234 | 0.039* |
| Model 2 | -0.220 | 0.043* |
| Model 3 | -0.228 | 0.042* |
|  | Log OC (ng/ml) | |
|  | β | P |
| Model 1 | -0.222 | 0.052 |
| Model 2 | -0.188 | 0.087 |
| Model 3 | -0.195 | 0.085 |

Model 1: adjusted for gender

Model 2: model 1 + HbA1c

Model 3: model 2 + TDD/kg and duration of diabetes

^*^ Statistically significant (P < 0.05).

β, standard partial regression coefficient; BMI, body mass index; ucOC, undercarboxylated osteocalcin; OC, osteocalcin; HbA1c, glycated hemoglobin; TDD/kg, body weight adjusted total daily dose of insulin.
